# Detecting Tuberculosis in Prisons: Switching Off the Disease at Its Source

**DOI:** 10.1093/cid/ciaa139

**Published:** 2020-02-17

**Authors:** Marc Woodman, Louis Grandjean

**Affiliations:** Department of Infection, Immunity and Inflammation, Institute of Child Health, University College London, London, UK

**Keywords:** tuberculosis, prisons, diagnosis, cost-effectiveness, algorithm

**(See the Major Article by Santos et al on pages 771–7.)**

*Mycobacterium tuberculosis* is estimated to have killed 1 billion people over the last 200 years [1] and remains the worlds’ most deadly human pathogen [2].

In order to improve upon the current 2% annual decline in tuberculosis incidence and get anywhere near the 90% by 2035 reduction target of the World Health Organization (WHO) “End Tuberculosis Strategy” [3], tuberculosis must be stopped at source.

There are few sources of tuberculosis worthier of intervention than tuberculosis disease arising in prisons. In resource-poor settings, the incidence of tuberculosis is on average 23 times greater inside prisons than in the surrounding community [4]. The incidence in the Brazilian prisons in this study was 3900 per 100 000, 100 times that of the general population. Unless tuberculosis disease is diagnosed and treated within the prison, on release, prisoners are more likely to return to high-risk transmission networks and fuel the epidemic further [5]. Prison settings often compound the problem by facilitating transmission through overcrowding, inadequate ventilation, incomplete treatment, late case detection, and high prisoner turnover [6]. Thus, the case for prison intervention is compelling.

It is naive to think that tuberculosis disease is contained within the walls of the prison. As a case in point, Sarita Colonia prison in the province of Callao, Peru, according to publicly available figures is overcrowded by 483% [7]. In 2018, it was shown that living in close proximity to the prison significantly increased the probability of sharing identical tuberculosis pathogen genotypes with those inside the prison [8]. Prison intervention will therefore diminish the incidence of tuberculosis cases in the surrounding community. Arguably even more concerning, because of prisoner exchange between countries, new cases of identical transmitted strains from Sarita Colonia prison in Peru have now emerged in both Florence and Madrid [9]. Therefore, prison intervention may even act to contain international tuberculosis spread.

Although much research time and many publications have focused on the benefits of intervening in prisons to diagnose and treat tuberculosis, there remains a lack of evidence to determine which—of all the options available—is the best to employ [10]. Santos and coauthors in this issue of *Clinical Infectious Diseases* present the results of an ambitious yet well-delivered study that helps to address the gaps in this important field of research.

The authors intensively and prospectively screened consenting prisoners in 3 Brazilian prisons for tuberculosis using symptom screening, GeneXpert, sputum culture, and chest radiography with computer-aided detection for tuberculosis (CAD4TB). They then retrospectively applied 4 alternative less comprehensive screening algorithms to the data to determine which was most cost effective per case detected. They found that 84% of tuberculosis cases were detectable by a single sputum sample for Xpert *M. tuberculosis*/rifampicin (MTB/RIF), and that systematic screening with this method had a cost per case of US$234. By comparison, symptom screening had a similar cost per case detected at US$235 but missed twice as many cases, whereas algorithms involving chest X-ray screening were more expensive and did not increase overall yield compared to testing with sputum Xpert MTB/RIF alone.

This is a clear, well written paper drawn from research undertaken in extremely difficult field conditions that has generated clinically applicable results. It demonstrates that the most sensitive and effective algorithm—of those that were tested—to detect tuberculosis disease in prisons is to apply GeneXpert to any prisoner who is able to produce sputum. Considering the challenging environment that the authors were working in, the screening participation rate of 89.9% is impressive and adds to the generalizability of the study.

Only 31% of patients were able to produce a sputum sample, which, as the authors conclude themselves, likely underestimates the true burden of tuberculosis disease.

This raises the question of whether interferon gamma release assays and/or tuberculin skin testing together with chest radiography could have a role in detecting these cases that were potentially missed. Although many studies have demonstrated the utility of GeneXpert as a point of care test and it is therefore expected that it would improve screening algorithms wherever it is applied, there are few studies that examine its use head to head with other algorithms in prisons.

Overall, the merit of this paper rests on the quality of its intensive screening strategy and the fact that this was applied to all prisoners in an extremely high burden setting. It clearly demonstrates the utility of GeneXpert in prisons—one of the major sources of new tuberculosis disease—and argues correctly for the implementation of this test in similar settings worldwide. If the WHO End TB targets are to be achieved, then well-informed, well-funded, and widespread scaling-up tuberculosis control in prisons is a good place to start.

## Notes

***Financial support.*** L. G. is supported by the Wellcome Trust (grant number 201470/Z/16/Z).

***Potential conflicts of interest.*** The authors: No reported conflicts of interest. Both authors have submitted the ICMJE Form for Disclosure of Potential Conflicts of Interest.
